# Decision-Making Under Risk, but Not Under Ambiguity, Predicts Pathological Gambling in Discrete Types of Abstinent Substance Users

**DOI:** 10.3389/fpsyt.2018.00239

**Published:** 2018-06-05

**Authors:** Michael J. Wilson, Jasmin Vassileva

**Affiliations:** ^1^Neuropsychology Section, VA Maryland Health Care System, Mental Health Service Line, Baltimore, MD, United States; ^2^Department of Psychiatry, Institute for Drug and Alcohol Studies, Virginia Commonwealth University, Richmond, VA, United States

**Keywords:** gambling, decision-making, substance dependence, risk-taking, impulsivity

## Abstract

This study explored how different forms of reward-based decision-making are associated with pathological gambling (PG) among abstinent individuals with prior dependence on different classes of drugs. Participants had lifetime histories of either “pure” heroin dependence (*n* = 64), “pure” amphetamine dependence (*n* = 51), or polysubstance dependence (*n* = 89), or had no history of substance dependence (*n* = 133). Decision-making was assessed via two neurocognitive tasks: (1) the Iowa Gambling Task (IGT), a measure of decision-making under ambiguity (i.e., uncertain risk contingencies); and (2) the Cambridge Gambling task (CGT), a measure of decision-making under risk (i.e., explicit risk contingencies). The main effects of neurocognitive performance and drug class on PG (defined as ≥3 DSM-IV PG symptoms) as well as their interactional effects were assessed via multiple linear regression. Two CGT indices of decision-making under risk demonstrated positive main effects on PG. Interaction effects indicated that the effects of decision-making under risk on PG were largely consistent across participant groups. Notably, a linear relationship between greater CGT Risk-Taking and PG symptoms was not observed among amphetamine users, whereas IGT performance was selectively and positively associated with PG in polysubstance users. Overall, results indicate that reward-based decision-making under risk may represent a risk factor for PG across substance users, with some variations in these relationships influenced by specific class of substance of abuse.

## Introduction

Pathological gambling (PG)^1^ is an addictive disorder characterized by recurrent patterns of compulsive gambling behavior that is associated with psychosocial burdens including financial debt, loss of productivity, legal difficulties, and psychiatric morbidity (1–4). PG represents a public health crisis, with point prevalence rates in North America and Western Europe of 1–6% (5). Although recreational gambling has reached unprecedented levels of popularity in Eastern Europe, very few studies have examined the corresponding prevalence of PG (6, 7) with epidemiological data indicating notable rates of PG in Hungary (3.3%) (7), Romania (7%) (8), and Lithuania (13–15%) (9). Further research is needed to better understand the cognitive mechanisms mediating the relationship between PG and substance use disorders (SUD) in Eastern Europe (10–13).

SUD and PG are frequently comorbid, with lifetime prevalence rates of alcohol and illicit SUD among pathological gamblers estimated at 73 and 38%, respectively (3). Similarly, up to 30% of substance users report having gambling problems (14, 15). Symptom overlap between PG and SUD includes tolerance, withdrawal, inability to maintain self-control, damage to significant relationships, commission of illegal acts to support compulsions, and persistence of compulsions despite negative consequences (16, 17). Recent studies utilizing neurochemical (18, 19), structural neuroimaging (20, 21), and functional neuroimaging methods (22–24) indicate shared neurobiological substrates between PG and SUD, which also share similar psychiatric comorbidity. Based on this evidence and in recognition of the serious public health challenges posed by this condition, PG was recently included in DSM 5 as Gambling Disorder (16, 25).

Neurocognitive aspects of impulsivity have been identified as core mechanisms underlying both PG and SUD (26). Neurocognitive dimensions of impulsivity are broadly conceptualized as: (1)“*action impulsivity,”* a.k.a “*rapid response impulsivity,”* associated with deficits in response inhibition, involving the inhibition or cancellation of prepotent or ongoing motor responses (27); and (2) “*choice impulsivity,”* measured with tasks of decision making or delay discounting and manifested as compromised ability to make decisions in line with long-term goals (28). One of the most salient aspects of choice impulsivity is impaired reward-based decision making, resulting in emotionally-mediated choices characterized by a preference for immediate rewards despite long-term negative consequences or greater benefit of delayed rewards (29, 30). Recent studies have identified at least two distinct forms of reward-based decision making: (a) *decision-making under risk*, measured by performance on tasks with explicit outcome probabilities; and (b) *decision-making under ambiguity*, measured by performance on tasks with implicit outcome probabilities. Both forms of decision-making are mediated by neural systems that are often dysregulated in individuals with PG or SUD, including the ventromedial prefrontal cortex (vmPFC), insular cortex, striatum, amygdala and parietal cortex (31–34). Substance dependent individuals (SDIs) often show impaired performance on measures of reward-based decision-making; for example, opiate use (35), methamphetamine use (36), and polysubstance use (37) have all been associated with impaired IGT performance relative to healthy controls. Pathological gamblers show equivalent impairments to SDIs on tasks of reward-based decision-making, reflection impulsivity, and future planning (38, 39). PG has been associated with impaired performance on the Iowa Gambling Task (IGT), an ecologically valid measure of decision-making under ambiguity that is sensitive to impaired decision-making among both individuals with vmPFC lesions and individuals with SUD (40, 41). PG has also been associated with impaired decision-making under risk (42, 43) as well as higher discounting of delayed rewards—a form of choice impulsivity frequently observed within the context of substance dependence (44–46).

Different drug classes, such as opiates and stimulants, have unique pharmacological properties, which may lead to differential neurocognitive and behavioral impulsivity profiles (30, 47, 48). For example, primary stimulant users have demonstrated greater levels of impairment on tasks of decision-making under ambiguity relative to primary opiate users (49–53). In contrast, several studies have failed to identify differences in decision-making under risk across groups of opiate, stimulant, and polysubstance users relative to controls (35–37, 47–58), though other findings have indicated differential decision-making performance between users of distinct classes of substances (59–62). Several factors may influence the variability of findings regarding decision-making within specific classes of substance users, including widespread histories of lifetime polysubstance dependence within research samples that ostensibly represent primary users of a specific drug class [see Discussions in (63, 64)]. For example, in one study reporting equivalently impaired IGT performance between groups of purported MDMA and polysubstance users (65), the MDMA user group also had notably higher levels of cocaine, hallucinogen, and sedative/hypnotic use relative to the polysubstance users. Further research that more precisely controls for history of substance dependence is therefore necessary to delineate associations between chronic use of specific pharmacologic drug classes and reward-based decision-making.

The goal of the present study is to explore relationships between two different types of reward-based decision-making: under risk and under ambiguity, and symptoms of PG in abstinent users of different classes of drugs, focusing on opiates and stimulants. Despite the high rates of comorbidity between substance use and gambling disorders, and the established importance of impaired reward-based decision-making in both types of disorders, we are not aware of previous studies that have comparatively examined associations between PG and different forms of reward-based decision-making across users of different drug classes. Notably, the current study examined individuals recruited from the Eastern European country of Bulgaria, and is among the first empirical efforts to examine neurocognitive mechanisms that may underlie PG in this population.

We examined whether differential relationships exist between PG symptoms and performance on the IGT, a neurocognitive measure of decision-making under ambiguity, and the Cambridge Gambling Task (CGT), a neurocognitive measure of decision-making under risk. Both tasks have proven sensitive to deficits in reward-based decision-making (66, 67) but differ in task demands. Successful performance on the IGT requires the integration of multiple neurocognitive functions, including learning, working memory, and reversal learning, whereas the CGT is a relatively pure measure of reward-based decision-making with explicit outcome probabilities. Therefore, a selective association of IGT performance with PG may indicate that cognitive complexity and situational ambiguity are candidate moderators of the relationship between impulsivity and PG in this population. Conversely, selective associations between CGT performance and PG may indicate that impairment in basic neurocognitive functions is less likely to contribute to the relationship between decision-making and PG.

Few studies to date have examined the influence of different classes of drugs on the associations of reward-based decision-making and PG. One recent study Zois et al. (43) found that, relative to controls, individuals with PG demonstrated impaired decision-making under risk that was equivalent across subgroups of pathological gamblers with no comorbid substance dependence, comorbid nicotine dependence only, and comorbid alcohol and nicotine dependence. Given that both PG and SUD have previously been associated with roughly equivalent deficits in both decision-making under risk and decision-making under ambiguity, we espouse the conservative hypothesis that performance on both tasks of reward-based decision-making will selectively predict PG symptoms across all drug classes. Given the high rates of comorbidity of PG and SUD in previous research samples, we additionally hypothesize that a history of dependence on any type of drug will be significantly associated with PG symptoms in our participants.

## Materials and methods

Participants were recruited in Sofia, Bulgaria as part of a larger study on impulsivity conducted at the Bulgarian Addictions Institute. The study was advertised through flyers placed in community substance abuse clinics and social venues including night clubs, bars and cafes, as well as through the study's web page and Facebook page. Participants were screened via telephone or in-person by structured interview assessing basic medical and substance use histories. All participants provided informed consent.

The study protocol consisted of two 3.5-h assessment sessions. All assessment instruments were translated into Bulgarian and back-translated into English. Neurocognitive assessments (see below) had virtually no language components. Participants were paid 80 Bulgarian Leva (≈US$50) for their participation in the study. All study procedures were approved by the Institutional Review Boards of Virginia Commonwealth University and the Medical University—Sofia on behalf of the Bulgarian Addictions Institute.

Study inclusion criteria were: (a) age of 18–50 years; (b) completion of minimum 8th grade education; (c) estimated IQ > 75 on Raven's Progressive Matrices (68); (d) no history of significant neurologic/neuropsychiatric illness; (e) no history of penetrating head injury or closed head injury with loss of consciousness > 30min; (f) no current mania or major depression; (g) negative breathalyzer test for alcohol and negative rapid urine toxicology screen for opiates, cannabis, amphetamines, methamphetamines, benzodiazepines, barbiturates, cocaine, MDMA, and methadone; (h) no current opioid substitution therapy.

### Assessment of substance use history

Detailed substance use histories were obtained using the substance abuse module of the Structured Clinical Interview for DSM-IV-Axis I Disorders (69). Inclusion criteria for substance users included lifetime history of DSM-IV dependence on either heroin or amphetamines. Inclusion criteria for healthy controls included no history of dependence on alcohol or other substances of abuse. All participants were confirmed as HIV-seronegative by rapid HIV testing.

### Assessment of pathological gambling

Lifetime PG symptoms were indexed using the gambling subscale (70) of the Addiction Severity Index-Lite [ASI-L; (71)], a semi-structured interview assessing history of substance use and related activities—including gambling—over the past 30 days and lifetime. ASI-L assessments were completed by a trained research assistant who conducted semi-structured interviews with participants. Participant responses to ASI-L gambling questions were cross-referenced with DSM-IV PG criteria (72). Total number of PG symptoms reported was tabulated as a dimensional symptom count variable.

### Assessment of reward-based decision-making

#### Iowa gambling task

The IGT (66) is a computerized task of decision-making under uncertainty which involves learning task contingencies by trial-and-error. Participants are presented with four decks of cards and are instructed to select cards with the goal to maximize earnings. Decks A and B are associated with higher rewards but also higher occasional penalties, while Decks C and D yield lower rewards but also lower occasional penalties. Choosing cards from decks C and D is a more advantageous long-term strategy typically not acquired by individuals with dysregulated vmPFC functioning (66, 67, 73) including individuals with SUD (59, 62). The IGT performance measure used in the analyses was the “net score” comprised of the total number of advantageous choices minus the total number of disadvantageous choices.

#### Cambridge gambling task

The CGT is a computerized task from the Cambridge Neuropsychological Test Automated Battery [CANTAB; (74)] designed to assess decision-making and risk-taking under explicit risk conditions. The examinee is presented with 10 boxes, each colored red or blue, and is instructed to guess whether a yellow token is hidden under a red or a blue box. The ratios of red: blue boxes vary from 1:9 to 9:1 in pseudorandom order. Thus, unlike the IGT, the odds of guessing correctly are presented explicitly to examinees by varying the ratios of colors among boxes that may contain the hidden token. Participants earn points based on correct performance and are asked to bet some proportion of their points for that trial (between 5 and 95%) on the certainty of their decision by selecting from an array of possible bets presented in ascending and descending sequences.

The CGT provides six performance indices: (a) Overall Proportion Bet: the average proportion of points risked over all trials; (b) Deliberation Time: the latency from the presentation of the colored boxes to bet choice; (c) Risk-Taking: the proportion of points risked when selecting the more likely outcome; (d) Quality of Decision-Making: the tendency to bet on the more likely outcome; (e) Risk Adjustment: betting more when odds are better and less when odds are poorer; and (f) Delay Aversion: the tendency to bet large amounts earlier (i.e., to bet more impulsively) when bet amounts are presented in ascending order (i.e., from 5 to 95%) rather than in descending order (i.e., from 95 to 5%).

### Statistical plan

A simultaneous-entry multiple linear regression model was computed to examine main effects of reward-based decision-making and drug class on PG symptoms, as well as decision-making × drug class interactions. Predictor variables included IGT net score and measures of CGT performance including Delay Aversion, Deliberation Time, Quality of Decision-Making, Risk Adjustment, and Risk-Taking. The Overall Proportion bet parameter from CGT was excluded from analyses due to high inter-correlation with CGT Risk-Taking (*r* = 0.97, *p* < 0.001); all other CGT parameters were judged to have sufficiently low inter-correlations (*r*'s 0.02–0.43) for simultaneous entry into the regression model. Categorical drug class variables were created via dummy-coding (with control participants used as a reference group). Statistical significance was set at *p* ≤ 0.05. Skew and kurtosis of continuous variables were inspected and neurocognitive variables were found to be normally distributed.

The dimensional count of PG symptoms was skewed (kurtosis = 3.88, *SE* = 0.281) and could not be corrected by transformation due to the absence of PG in most participants. Therefore, a categorical variable indicating the presence of absence of PG (i.e., coding = 1 if PG present; coding = −1 if PG not present) was used as the dependent variable for the regression model. The number of participants who met full DSM-IV criteria for PG diagnosis (i.e., ≥5 symptoms) was low (*n* = 38, 11%); therefore, a criterion of ≥3 DSM-IV symptoms was used to define the presence of PG, in accordance with methods from prior published reports (75–78). Additionally, partial Spearman rank correlations examining associations between neurocognitive decision-making and PG symptom counts were calculated within each participant group, in order to explore the relationship of neurocognitive decision-making with severity of PG symptoms across users of different classes of drugs.

## Results

### Participant characteristics

Participant demographic characteristics are presented in Table 1. The total sample (*N* = 337) consisted of 133 healthy controls and 204 substance users. Substance users were designated as “pure” heroin dependent (*n* = 64); “pure” amphetamine dependent (*n* = 51); or polysubstance dependent (*n* = 89). Length of abstinence was recorded via self-report. On average, polysubstance users reported meeting DSM-IV criteria for heroin and amphetamine dependence approximately one and one-and-a-half years prior to the study, respectively. In contrast, heroin users reported meeting criteria for heroin dependence ~3 years prior to the study, while amphetamine users reported meeting criteria for amphetamine dependence ~2 years prior to the study (see Table 1). Most substance users (64%, *n* = 131) met criteria for protracted (i.e., >1 year) abstinence from substance dependence. No heroin users met criteria for current substance dependence, while one amphetamine user (2%) met criteria for current amphetamine dependence. Among polysubstance users, five (10%) met criteria for current amphetamine dependence, four (8%) met criteria for current alcohol dependence, and four (8%) met criteria for current cannabis dependence.

**Table 1:** Participant group characteristics.

	**Controls (*n* = 133)**	**Heroin (*n* = 64)**	**Amphetamine (*n* = 51)**	**Polysubstance (*n* = 89)**	**Sig. Testing**
Sex (*# females, %*)	40^a^ [30]	15^b^ [23]	17^b^ [33]	13^b^ [15]	χ^2^ = 8.9*
Age (*M, SD*)	25.21^a^ (5.81)	29.30^b^ (4.57)	23.20^c^ (3.85)	26.35^a^ (522)	*F* = 14.7*
Years of education	13.32 (2.75)	12.78 (2.39)	13.04 (2.20)	13.02 (2.13)	*F* = 0.7
Estimated IQ	107 (15)	103 (13)	110 (11)	106 (14)	*F* = 2.3
# PG symptoms	0.68^a^ (1.97)	1.44^b, c^ (2.54)	0.67^a, b^ (1.97)	1.67^c^ (2.87)	*F* = 3.8*
# Weeks since last gambled	80^a^ (137)	269^b^ (253)	76^a^ (108)	141^a^ (195)	*F =* 8.70*
**YEARS OF SUBSTANCE USE (*****M, SD*****)**					
Heroin	0	7.09^a^ (3.30)	0	3.72^b^ (4.95)	*t* = 5.0*
Amphetamine	0.62^a^ (2.08)	0.14^a^ (0.87)	3.66^b^ (2.31)	2.70^b^ (2.97)	*F* = 35.9*
Alcohol	9.12^a^ (5.61)	11.50^b^ (5.44)	8.01^a^ (3.77)	10.46^b^ (5.50)	*F* = 5.4*
Other	2.03^a^ (3.93)	10.61^b^ (3.27)	6.90^c^ (3.29)	10.26^b^ (4.27)	*F* = 93.4*
**DSM-IV LIFETIME SUBSTANCE DEPENDENCE (*****#, %*****)**					
Heroin	0	64^a^ [100]	0	42^b^ [48]	χ^2^ = 239.9*
Amphetamine	0	0	51^a^ [100]	54^b^ [61]	χ^2^ = 257.8*
Alcohol	0	0	0	27 [30]	–
Cannabis	0	0	0	68 [76]	–
Cocaine	0	0	0	8 [9]	–
Sedatives	0	0	0	9 [10]	–
**YEARS SINCE LAST MET DEPENDENCE CRITERIA (*****M, SD*****)**					
Heroin	–	3.18^a^ (2.68)	–	0.75^b^ (1.37)	*t* = 5.08*
Amphetamine	–	–	2.23 (1.75)	1.61 (1.93)	*t* = 1.48
**REWARD-BASED DECISION-MAKING PERFORMANCE (*****M, SD*****)**					
CGT delay aversion	0.34 (0.23)	0.42 (0.21)	0.35 (0.19)	0.38 (0.22)	*F* = 0.15
CGT delib time (ms)	2,356 (712)	2,318 (692)	2,507 (697)	2,384 (763)	*F* = 0.54
CGT qual. decisions	0.87 (0.13)	0.85 (0.16)	0.86 (0.12)	0.86 (0.13)	*F* = 0.63
CGT risk adjustment	0.94 (0.94)	0.86 (.91)	0.93 (0.86)	0.79 (0.74)	*F* = 0.66
CGT risk taking	0.62 (0.12)	0.59 (014)	0.60 (0.14)	0.62 (014)	*F* = 0.58
Iowa gambling task	3.30 (24.06)	1.56 (28.68)	3.65 (24.19)	−2.0 (26.53)	*F* = 0.47

Discordant superscripts indicate sig. differences; *p < 0.05; CGT, Cambridge Gambling Task.

PG symptoms were reported by 20% of healthy controls (*n* = 27; # symptoms range = 1–9); 31% of heroin users (*n* = 20; # symptoms range = 1–9); 14% of amphetamine users (*n* = 7; # symptoms range 1–8); and 30% of polysubstance users (*n* = 27; # symptoms range 1–10). Out of all participants, 18% (*n* = 60) met criteria for PG (i.e., ≥3 DSM-IV PG symptoms). Criteria for PG (i.e., ≥3 PG symptoms) were met by 13% of controls (*n* = 17), 23% of heroin users (*n* = 15), 8% of amphetamine users (*n* = 4), and 26% of polysubstance users (*n* = 23). Average recency of gambling behavior across the full sample was 80 weeks (*SD* = 137). Heroin users reported a significantly longer duration of time since they last gambled compared to all other participants (*p*'s < 0.005); no other significant between-group differences in recency of gambling were observed (*p*'s > 0.10).

Significant between-group differences were observed for dimensional PG symptoms [*F*_(3, 321)_ = 3.81, *p* = 0.010, η^2^_*p*_ = 0.034]. Amphetamine users (*M* = 0.67, *SD* = 1.97) reported equivalent PG symptom levels with healthy controls (*M* = 0.68, *SD* = 1.75, *p* = 0.982), while polysubstance users reported equivalent PG symptom levels with heroin users (*M* = 1.44, *SD* = 2.54, *p* = 0.545). Polysubstance users (*M* = 1.67, *SD* = 2.87) reported more PG symptoms than healthy controls (*p* = 0.002) and amphetamine users (*p* = 0.016). Finally, heroin users reported more PG symptoms than healthy controls (*p* = 0.031) and demonstrated a trend toward more PG symptoms than amphetamine users (*p* = 0.078).

### Selection of covariates

Education and estimated IQ were examined as potential covariates for analyses, given that these demographic variables did not systematically vary as a function of participant group (see Table 1 for omnibus tests of demographic variables). A series of bivariate correlations was conducted examining zero-order associations between these potential covariates, neurocognitive decision-making indices, and PG symptoms. IQ was not significantly associated with the presence of PG (*p* = 0.109) and was not included as a covariate for regression analyses. In contrast, IQ was selected as a covariate for partial correlation analyses based on significant correlations of IQ with PG dimensional symptoms (*r* = −0.12, *p* = 0.026), total IGT performance (*r* = 0.20, *p* < 0.001), and several CGT indices (Delay Aversion *r* = −0.27, *p* < 0.001; Quality of Decision-Making *r* = 0.19, *p* = 0.001; Risk Adjustment *r* = 0.29, *p* < 0.001). Education was not correlated with PG symptom presence or symptom count (*p*'s > 0.15) and was therefore not included as a covariate in either regression or partial correlation analyses.

### Effects of drug class and reward-based decision-making on the presence of pathological gambling symptoms

Main effects from the multiple linear regression analysis are presented in Table 2 and interaction effects are presented in Table 3. The overall regression model was associated with significant variance in PG (*R*^2^ = 0.17, *F* = 2.2, *p* = 0.001). A significant main effect of amphetamine dependence on PG was observed (β = 1.4, *p* = 0.052). A marginally significant trend for a main effect of polysubstance dependence on PG was also observed (β = 1.42, *p* = 0.061). In contrast, there was no main effect of heroin dependence on PG (*p* = 0.949).

**Table 2:** Main effects from regression model examining the effects of reward-based decision-making, drug class, and their interactions on pathological gambling.

	**β**	***t***	***p***
**CLASS OF SUBSTANCE DEPENDENCE**			
Heroin	0.047	0.064	0.949
Amphetamine	1.34	1.95	0.052
Polysubstance	1.42	1.88	0.061
**NEUROCOGNITIVE DECISION-MAKING**			
Iowa gambling task	−0.146	−1.51	0.131
CGT quality of decisions	−0.041	−0.407	0.685
CGT risk adjustment	0.270	2.31	0.022
CGT delay aversion	0.205	1.58	0.114
CGT deliberation time	0.100	1.02	0.308
CGT risk taking	0.316	2.60	0.010

*CGT, Cambridge Gambling Task*.

**Table 3:** Interaction effects from regression model examining the effects of reward-based decision-making, drug class, and their interactions on pathological gambling.

	**β**	***t***	***p***
**HEROIN USER INTERACTIONS**			
Iowa gambling task	0.006	0.078	0.938
CGT quality of decisions	0.021	0.051	0.959
CGT risk adjustment	−0.002	−0.018	0.985
CGT delay aversion	−0.054	−0.290	0.772
CGT deliberation time	−0.055	−0.240	0.811
CGT risk taking	0.124	0.353	0.724
**AMPHETAMINE USER INTERACTIONS**			
Iowa gambling task	0.003	0.047	0.963
CGT quality of decisions	−0.191	−0.381	0.704
CGT risk adjustment	−0.117	−0.942	0.347
CGT delay aversion	−0.191	−0.381	0.704
CGT deliberation time	−0.286	−1.16	0.246
CGT risk taking	−0.761	−2.11	0.036
**POLYSUBSTANCE USER INTERACTIONS**			
Iowa gambling task	0.224	3.04	0.003
CGT quality of decisions	−0.754	−1.70	0.091
CGT risk adjustment	−0.022	−0.203	0.839
CGT delay aversion	−0.203	−1.21	0.226
CGT deliberation time	0.000	0.002	0.999
CGT risk taking	−0.359	−0.967	0.334

*CGT, Cambridge Gambling Task*.

A significant main effect was observed for CGT Risk-Taking (β = 0.32, *p* = 0.010), indicating that across all participants, wagering more points on a potentially favorable outcome during CGT trials was associated with PG. A significant main effect of CGT Risk Adjustment was also observed (β = 0.27, *p* = 0.022), indicating that advantageously adjusting betting amounts in response to CGT trial contingencies was associated with PG. There were no observed main effects of CGT Delay Aversion, Deliberation Time, or Quality of Decision-Making (*p*'s > 0.10). IGT performance also had no significant main effect on PG (*p* = 0.131).

A significant interaction of CGT Risk-Taking and amphetamine dependence was observed (β = −0.76, *p* = 0.036); follow-up examination of the marginal means of this interaction indicated that the positive association of CGT Risk-Taking with PG was not observed among amphetamine users. No other significant CGT × drug class interactions were observed (*p*'s ≥ 0.09). A significant polysubstance dependence × IGT interaction was observed (β = 0.22, *p* = 0.003), indicating that more advantageous performance on the IGT was positively and selectively associated with PG among polysubstance users.

### Within-group associations of neurocognitive decision-making and dimensional pathological gambling symptoms

Results of partial Spearman's correlations examining relationships between PG dimensional symptoms and measures of decision-making across all participants revealed that more advantageous Risk-Taking on the CGT was associated with higher levels of PG symptoms, ρ = 0.30, *p* = 0.001. A selective association of IGT performance with PG dimensional symptoms was observed within polysubstance users, indicating that for this subgroup of participants, more advantageous decision-making under ambiguity was associated with higher levels of PG symptoms, ρ = 0.27, *p* = 0.019. No significant partial correlations were observed between decision-making performance on either the CGT or IGT and PG dimensional symptoms in heroin users or amphetamine users (*p*'s > 0.10), a finding which may be influenced by reduced statistical power of our within-group analyses.

## Discussion

This study investigated the relative contributions of decision-making under ambiguity and decision-making under risk to PG within abstinent heroin, amphetamine, and polysubstance users. We hypothesized that both forms of reward-based decision-making would predict PG symptoms across all drug users, and that history of dependence on any drugs of abuse would be associated with PG symptoms. Our results indicated that histories of amphetamine and polysubstance dependence, but not heroin dependence, were selectively associated with the presence of PG. Risk Adjustment on the CGT, an index of adaptive decision-making under explicit risk conditions, proved to be an effective predictor of PG across all participants. Similarly, Risk-Taking on the CGT was associated with PG across healthy controls, heroin users, and polysubstance users, but not among amphetamine users. By comparison, decision-making under ambiguity as indexed by the IGT, was associated with PG only among polysubstance users.

The CGT parameters which emerged as significant predictors of PG encapsulate several aspects of reward-based decision-making. Higher levels of risk adjustment were associated with PG across all participants. Engaging in risk adjustment may represent an advantageous approach to the CGT, but may also indicate a more general tendency to make relatively risky bets when the odds are perceived as more favorable. Therefore, risk adjustment may potentially result in greater monetary losses in real-life gambling scenarios, or may suggest a greater susceptibility to the reinforcing effects of gambling behavior. Greater risk-taking on the CGT was also associated with PG for most participants, suggesting that another relatively advantageous task strategy (i.e., wagering more when the more likely/advantageous outcome is selected) may also translate to a greater tendency to engage in PG in real-world contexts. This assumption is supported by findings from our correlational analyses indicating that CGT Risk-Taking was selectively associated with dimensional PG severity across all participants.

Higher risk-taking on the CGT was selectively dissociated from the presence of PG among amphetamine users in our sample. These between-group variations in the association between neurocognitive aspects of decision-making and PG may be influenced by differences in component decision-making processes among users of different drug classes (79). Notably, computational modeling analyses of IGT performance among a subset of abstinent substance users drawn from the same population as our current sample (80) indicate that abstinent amphetamine users demonstrate increased reward sensitivity, while abstinent opiate users evidence decreased loss aversion. Given that previous research has linked deficits in pre-choice emotional appraisal and feedback sensitivity to impairments in reward-based decision-making (81, 82), the predictive utility of reward-based decision-making paradigms may be limited in our sample of abstinent amphetamine users due to increased reward sensitivity. High reward sensitivity may lead to more accurate risk-reward appraisals, and possibly lead to less risky behavior in real-world gambling scenarios that have higher potential monetary rewards (and thus higher reward salience) than can be attained in the laboratory setting. In contrast, decreased loss aversion in our sample of abstinent heroin users may contribute to deficient appraisal in situations of high reward salience, leading to more comparable cross-situational reward-based decision-making for both laboratory task performance and real-world gambling scenarios. Consistent with this hypothesis, loss aversion in opiate users has been negatively correlated with psychopathy (80), a construct in which the central neurocognitive finding is deficient avoidance learning (83, 84) and which is associated with elevated risk for addictive behaviors (85, 86). Future research inquiries from our group will examine how computationally derived IGT parameters of reward sensitivity and loss aversion are related to PG symptoms and CGT performance in our current samples of abstinent substance users.

It is notable that although IGT performance was not associated with PG across most participants, a selective positive association of IGT performance and PG was observed among polysubstance users. Given the ambiguity of reward contingencies on the IGT relative to the CGT, the observed pattern of results suggest that relatively pure deficits in reward-based decision-making contribute to PG across all groups of substance users, whereas the impact of ambiguity on decision-making appears to only contribute to PG among polysubstance users. Polysubstance users may therefore be at greater risk for PG than users of a single drug class, due to evidence that both decision-making under risk and decision-making under ambiguity appear to influence PG among polysubstance users. Interestingly, a recent study conducted in Belgium (87) found that reward-based decision-making under ambiguity, but not under risk, was associated with PG symptoms among a sample of problem gamblers. This suggests that our sample of polysubstance users may more closely resemble individuals with primary gambling problems than our samples of heroin and amphetamine users. This impression is supported by evidence from our within-group correlational analyses indicating that IGT performance was selectively associated with severity of PG in polysubstance users. However, it should be noted that the IGT is not strictly a pure measure of risk-taking under ambiguity, as contingencies for this task may be probabilistically inferred over time, in contrast to risk-taking measure with truly randomized contingencies, such as the Balloon Analog Risk Task (88). Therefore, the mechanism driving the association between IGT performance and PG may not be strictly due to the effects of decision-making under ambiguity, and other alternative mechanisms of IGT performance (e.g., the higher cognitive complexity of this task relative to the CGT) could be examined in future investigations.

Several limitations should be noted regarding the current study. First, although participant groups were well-matched on key demographic variables including education level and estimated IQ, systematic group differences on other demographic variables (e.g., age) may have influenced results. Secondly, this study was cross-sectional, and future prospective studies will be required to definitively establish the directionality on the observed relationships between decision-making and PG. Third, relatively few amphetamine users in the current sample endorsed any symptoms of PG (*n* = 7, 14%), which may have influenced findings for this group. However, it is unknown whether or not the observed base rate of PG symptoms in this group is to be expected among populations of abstinent amphetamine users, due to a lack of targeted PG research within this subpopulation. Finally, substance dependence is a highly heterogeneous condition often associated with multiple comorbidities, which may influence variations in patterns of association between neurocognitive decision-making and risk behavior. For example, the current study does not address the question of whether decision-making performance moderates or mediates the influence of externalizing personality traits (e.g., trait impulsivity) on PG, and future studies by our group will examine this question directly.

Studies examining pathological gamblers [e.g., (87)] do not always specify the period of remission from substance use or compulsive gambling behavior. Thus, it is plausible that acute drug effects may have affected reward-based performance in these samples. Our findings raise the possibility that stage of the addiction cycle may influence the association between decision-making and PG, in addition to potential moderating factors of the nature of addiction (i.e., behavioral addiction vs. substance dependence, specific classes of drugs, mono- vs. polysubstance dependence). Comparative examinations of individuals with SUD, pathological gamblers without comorbid SUD, and dually diagnosed individuals at different stages of the addiction cycle will serve to further elucidate the influence of specific types of addiction on decision-making and gambling behavior.

## Ethics statement

This study was carried out in accordance with the recommendations of the Declaration of Helsinki. All subjects gave written informed consent prior to participation in the study. The protocol was approved by the Ethics Committees of Virginia Commonwealth University and Medical University—Sofia.

## Author contributions

MW designed the data analytic plan, conducted analyses, performed literature review, and wrote the manuscript and tables. JV designed the study, wrote the study research protocol, oversaw data collection, made contributions to the content of the manuscript, and provided proof-reading and editing for the manuscript. Both authors approve the final manuscript.

### Conflict of interest statement

The authors declare that the research was conducted in the absence of any commercial or financial relationships that could be construed as a potential conflict of interest.

## Footnotes

^1^We used DSM-IV criteria to identify clinically relevant gambling symptoms; therefore, we use the term “pathological gambling” throughout the paper, instead of “gambling disorder” as defined by the DSM-5.
